# Personal Information Protection and Privacy Policy Compliance of Health Code Apps in China: Scale Development and Content Analysis

**DOI:** 10.2196/48714

**Published:** 2023-11-14

**Authors:** Jiayi Jiang, Zexing Zheng

**Affiliations:** 1 Law School, Central South University, Changsha, China

**Keywords:** contact tracing, privacy policy, personal information protection, compliance, content analysis, COVID-19

## Abstract

**Background:**

Digital technologies, especially contact tracing apps, have been crucial in monitoring and tracing the transmission of COVID-19 worldwide. China developed health code apps as an emergency response to the pandemic with plans to use them for broader public health services. However, potential problems within privacy policies may compromise personal information (PI) protection.

**Objective:**

We aimed to evaluate the compliance of the privacy policies of 30 health code apps in the mainland of China with the Personal Information Protection Law (PIPL) and related specifications.

**Methods:**

We reviewed and assessed the privacy policies of 30 health code apps between August 26 and September 6, 2023. We used a 3-level indicator scale based on the information life cycle as provided in the PIPL and related specifications. The scale comprised 7 level-1 indicators, 26 level-2 indicators, and 71 level-3 indicators.

**Results:**

The mean compliance score of the 30 health code apps was 59.9% (SD 22.6%). A total of 13 (43.3%) apps scored below this average, and 6 apps scored below 40%. Level-1 indicator scores included the following: general attributes (mean 85.6%, SD 23.3%); PI collection and use (mean 66.2%, SD 22.7%); PI storage and protection (mean 63.3%, SD 30.8%); PI sharing, transfer, disclosure, and transmission (mean 57.2%, SD 27.3%); PI deletion (mean 52.2%, SD 29.4%); individual rights (mean 59.3%, SD 25.7%); and PI processor duties (mean 43.7%, SD 23.8%). Sensitive PI protection compliance (mean 51.4%, SD 26.0%) lagged behind general PI protection (mean 83.3%, SD 24.3%), with only 1 app requiring separate consent for sensitive PI processing. Additionally, 46.7% (n=14) of the apps needed separate consent for subcontracting activities, while fewer disclosed PI recipient information (n=13, 43.3%), safety precautions (n=11, 36.7%), and rules of PI transfer during specific events (n=10, 33.3%). Most privacy policies specified the PI retention period (n=23, 76.7%) and postperiod deletion or anonymization (n=22, 73.3%), but only 6.7% (n=2) were committed to prompt third-party PI deletion. Most apps delineated various individual rights: the right to inquire (n=25, 83.3%), correct (n=24, 80%), and delete PI (n=24, 80%); cancel their account (n=21, 70%); withdraw consent (n=20, 60%); and request privacy policy explanations (n=24, 80%). Only a fraction addressed the rights to obtain copies (n=4, 13.3%) or refuse advertisement of automated decision-making (n=1, 3.3%). The mean compliance rate of PI processor duties was only 43.7% (SD 23.8%), with significant deficiencies in impact assessments (mean 5.0%, SD 19.8%), PI protection officer appointment (mean 6.7%, SD 24.9%), regular compliance audits (mean 6.7%, SD 24.9%), and complaint management (mean 37.8%, SD 39.2%).

**Conclusions:**

Our analysis revealed both strengths and significant shortcomings in the compliance of privacy policies of health code apps with the PIPL and related specifications considering the information life cycle. As China contemplates the future extended use of health code apps, it should articulate the legitimacy of the apps’ normalization and ensure that users provide informed consent. Meanwhile, China should raise the compliance level of relevant privacy policies and fortify its enforcement mechanisms.

## Introduction

### Background

The COVID-19 pandemic has posed significant public health challenges worldwide, prompting many countries to adopt various digital strategies, including contact-tracing apps to monitor the transmission of the virus [1,2]. These technological innovations have allowed for an unprecedented level of information collection, aggregation, analysis, and dissemination [3], and have brought significant benefits, such as the ability to track cases, identify potential outbreaks, and inform public health interventions [4,5]. However, the extensive use and accelerated development of contact-tracing apps have raised concerns regarding individual privacy rights and personal information (PI) [6-9], with lasting and profound impacts on data governance and PI protection [10]. To address these concerns, proponents of these apps, including governments and developers, should follow legal guidelines concerning privacy principles and policy content [11-13].

Although the short-term use of contact-tracing apps may be justified given the public emergencies the pandemic caused, the legitimacy of long-term use after the pandemic should be assessed to enhance PI protection and improve data governance capabilities [14]. As the global pandemic situation has stabilized, some countries have evaluated the necessity of using contact-tracing apps and decided to shut down such services. In the United Kingdom, the National Health Service COVID-19 app closed down on April 27, 2023 [15], after preventing around 1 million cases, 44,000 hospitalizations, and 9600 deaths in its first year alone [16]. In India, the contact-tracing feature of the Aarogya Setu app has been disabled, and the contact-tracing data collected has been deleted [17]. Singapore’s government has been progressively rolling back its TraceTogether and SafeEntry platforms as the global pandemic situation stabilized, with all identifiable data collected via the two platforms wiped from their servers and databases [18].

In China, health code apps have been implemented as an essential tool for classifying citizens based on different transmission risk levels, quickly locating people who are potentially infected, and implementing control measures promptly [19-21]. These apps are designed to be a dynamic health certification that allows government agencies, employers, and communities to identify personal health risk levels and grants individuals the qualifications for mobility and work resumption [22]. Health code apps collect various types of sensitive PI. According to the Personal Health Information Code-Data Format (GB/T 38962-2020), PI collected to generate health codes includes the user’s identity (eg, name, contact information, and medical history), health (eg, body temperature and current symptoms), travel (eg, residence and geographical location), and health certificate (eg, health risk level, evaluation, and medical examination results), among other information. Sensitive PI collected to generate health codes flows from users to digital platforms, medical institutions, and governments in existing practices [23]. The collection of PI is susceptible to invasion, which can lead to discrimination and harm [24]. Therefore, the increasing risk of leaks and abuse of PI due to the aggregated storage of data collected to generate health codes has raised concerns about privacy violations from public powers [25,26].

Instead of shutting down health code apps in the postpandemic era, China has promoted the use of health code apps for a broader public health service scope [27]. Each citizen will have a dynamic electronic health file and a multifunctional health code by 2025 [28], which will act as a major index of personal health information in disease prevention, medical care, rehabilitation, and health management [29]. Meanwhile, health code apps will serve as strategic health and medical service platforms. This strategy will turn individual health data, such as medical records and biometrics, into critical assets to reinforce government administration and social management [30]. Such an extension has raised concerns regarding the normalization of health code apps in the postpandemic society and the routinization of expanded government power [31,32].

China has realized the importance and urgency of PI protection and established a regulatory framework. Some national voluntary standards have come into effect, in particular the Information Security Technology–Personal Information Specification (GB/T 35273-2020; PI Specifications), which laid out granular guidelines for how PI should be collected, used, and shared to operate health code apps [33]. In addition, the Personal Information Protection Law (PIPL), which came into force on November 11, 2021, guarantees the rights of individuals and places constraints on PI processors. PIPL is regarded as a milestone for regulating PI protection specifically [34].

The existing application and potential normalization of health code apps have presented significant challenges for PI protection [14,35,36]. However, the legal compliance of health code apps remains unclear. Experts, authorities, and users need to assess the risks of PI protection and determine the future of health code apps. Notably, several potential problems within the privacy policies of health code apps may compromise the effectiveness of legal protections for PI, including the readability of the privacy policies, extensive PI collection, multiple processing purposes, indeterminate storage duration, and ambiguous privacy policy content [12,13,37,38].

### Objective

In this study, we aimed to collect the privacy policies of health code apps developed by the provincial administrative regions in the mainland of China and assess the compliance of these privacy policies with the PIPL and PI Specifications from the information life cycle. We hope this study can contribute to the global discussion on balanced policies for PI protection in digital health initiatives in the postpandemic era, providing insights for policy makers, health code developers, and users across different countries while highlighting the importance of improving legal compliance and strengthening enforcement.

## Methods

We conducted a content analysis of the privacy policies of health code apps developed in 31 provincial administrative regions in the mainland of China and evaluated their compliance with the PIPL and PI Specifications.

### Ethical Considerations

Ethical review does not apply to our research because no experiments on human participants were completed.

### Apps Access and Privacy Policies Collection

We searched for health code apps developed by provincial administrative regions on August 24, 2023. We accessed the health code apps of various provincial administrative regions through the National Government Service Platform, a national digitally integrated platform of government services available on the WeChat mini-program (operated by Tencent) and Alipay (operated by Alibaba). We obtained and reviewed the full text of corresponding privacy policies as text files or screenshots from the WeChat mini-program, Alipay, and Baidu, a well-known Chinese search engine, between August 25 and September 6, 2023.

### Scale Development and Scoring

We used level-1 evaluation indicators based on the information life cycle as provided in the PI Specifications and the PIPL. These indicators encompassed the following stages: PI collection and use; PI storage and protection; PI sharing, transfer, disclosure, and transmission; PI deletion; general attributes; individual rights; and PI processor duties. We further elaborated these categories into 26 level-2 indicators and 71 level-3 indicators, each aligned with the specific provisions of the PIPL and PI Specifications. We provided brief explanations, example sentences, and corresponding references to provisions of the PI Specifications and the PIPL in Multimedia Appendix 1.

We assigned a score of 1 for each level-3 indicator if the privacy policy complied with the specific indicator and a score of 0 if it did not. Each level-3 indicator’s compliance rate was determined as the proportion of policies that scored “1” from the sample of 30 apps. The scoring rate of each level-2 indicator was the arithmetic mean of the scoring rates of all associated level-3 indicators. Likewise, the compliance rate of level-1 indicators was the mean of its corresponding level-2 rates, thus representing the overall compliance of each app at specific information life cycle stages. For each privacy policy, the aggregate of all level-3 indicators was calculated as a total score and converted into a percentage system as a final score to denote the overall compliance of a given policy. Two independent raters (JJ and ZZ) collaboratively assessed all 30 privacy policies between August 25 and September 6, 2023.

## Results

### Sample Collection

We accessed the health code apps of all 31 provincial administrative regions in the mainland of China and obtained the full text of 30 privacy policies, including 23 from WeChat, 3 from Alipay, and 4 sourced manually from Baidu. The privacy policy of the health code app of Chongqing City was unavailable on the referenced platforms or search engine. Notably, the health code apps for Heilongjiang Province and Qinghai Province lacked distinct privacy policies. Heilongjiang’s approach involved a tick box where users ensured the accuracy of information for COVID-19 prevention and control, while Qinghai integrated its privacy provisions within the user agreement. In addition, the health code apps of Ningxia Hui Autonomous Region and Tibet Autonomous Region used a common privacy policy template, differing only in the basic PI processor information.

### Compliance Evaluation

The overall compliance landscape among the 30 assessed privacy policies of health code apps presented a mixed picture. The mean compliance score of the 30 privacy policies was 59.9% (SD 22.6%). A total of 17 (56.7%) apps surpassed the mean score, while 13 (43.3%) apps fell below it.

The evaluation results on the privacy policies’ level-1 and level-2 indicators are listed in Table 1. The level-1 indicators were ranked from highest to lowest scores as follows: general attributes (mean 85.6%, SD 23.3%); PI collection and use (mean 66.2%, SD 22.7%); PI storage and protection (mean 63.3%, SD 30.8%); individual rights (mean 59.3%, SD 25.7%); PI sharing, transfer, disclosure, and transmission (mean 57.2%, SD 27.3%); PI deletion (mean 52.2%, SD 29.4%); and PI processors duties (mean 43.7%, SD 23.8%). The names and evaluation results of each app are listed in Multimedia Appendix 2.

**Table 1.** Compliance evaluation rates for level-1 and level-2 indicators in privacy policies.

Evaluation results on level-1 and level-2 indicators		Compliance rate (%), mean (SD)
**General attributes**		85.6 (23.3)
	PI^a^ processors and service	93.3 (21.3)
	Policy transparency	95.6 (18.7)
	Policy maintenance	74.2 (34.5)
**PI collection and use**		66.2 (22.7)
	Collection and use of general PI in service functions	83.3 (24.3)
	Collection and use of sensitive PI in service functions	51.4 (26.0)
**PI storage and protection**		63.3 (30.8)
	Storage security	65.8 (32.6)
	Security incidents	60.0 (35.9)
**PI sharing, transfer, disclosure, and transmission**		57.2 (27.3)
	Subcontracting of PI processing	46.7 (28.7)
	PI sharing and transfer	53.3 (32.4)
	Public disclosure	85.0 (32.0)
	Cross-border transmission	53.3 (38.6)
**PI deletion**		52.2 (29.4)
	Retention period	76.7 (42.3)
	Deletion and cessation	40.0 (27.1)
**Individual rights**		59.3 (25.7)
	Inquiry of PI	83.3 (37.3)
	Obtain copies of PI	13.3 (34.0)
	Correction of PI	80.0 (40.0)
	Deletion of PI	80.0 (40.0)
	Explanation regarding PI processing	80.0 (40.0)
	Consent withdrawal	31.7 (27.3)
	Deregistration	70.0 (45.8)
	Consent exception scenarios	63.3 (48.2)
**PI processor duties**		43.7 (23.8)
	PI protection officer disclosure	6.7 (24.9)
	Compliance audits	6.7 (24.9)
	Impact assessment procedures	5.0 (19.8)
	Request management	60.7 (30.3)
	Complaint management	37.8 (39.2)

a PI: personal information.

The privacy policies’ general attributes (mean 85.6%, SD 23.3%) scored high, which reflected their transparency and maintenance. Some level-2 indicators scored notably high, including PI processors and service (mean 93.3%, SD 21.3%) and policy transparency (mean 95.6%, SD 18.7%). These high scores indicated that most privacy policies identified who was responsible for processing PI and providing services. Policy maintenance was another strong area, with a score of 74.2% (SD 34.5%). Of the 30 apps, most appeared to be diligent in updating their policies, with 25 (83.3%) indicating they did so occasionally. Additionally, 80% (n=24) of the apps notified users about updates through various methods (eg, email or pop-up alerts). More than half of the privacy policies exceeded requirements by obtaining separate consent for specific policy changes (n=20, 66.7%) or by clearly marking the effective or updated dates (n=20, 66.7%). Specifically, 16 (53.3%) apps updated their privacy policies after the PIPL came into force. However, concerningly, 10 (33.3%) apps failed to mention either the effective date or the updated date of their policies, while 4 apps updated their privacy policies before the PIPL came into effect.

The scoring rate of collection and use of general PI (mean 83.3%, SD 24.3%) was high, which indicated that a majority of health code apps were attentive to articulating the collection and use of general PI in their privacy policies. The privacy policies of almost all apps delineated the purpose (n=29, 96.7%) and methods (n=29, 96.7%) of collecting and using general PI. A large number (n=28, 93.3%) of apps listed the types of PI collected and used, while 25 (83%) elaborated on the specific service functions that require such information. This suggested that users were generally well-informed about how their PI would be collected and used. However, a modest majority of the privacy policies differentiated between necessary PI and nonessential PI (n=19, 63.3%) and explained the consequences of failing to provide certain types of PI (n=20, 66.7%). This left room for improvement in ensuring that individuals fully understood which information was mandatory versus optional and the consequences of not providing it. By contrast, the scoring rate of collection and use of sensitive PI (mean 51.4%, SD 26.0%) was relatively low. Most privacy policies communicated the implications of processing sensitive PI (n=24, 80%) and required consent for collecting PI from minors (n=24, 80%). More than half of the privacy policies outlined protective measures (n=19, 63.3%) and specified the purposes (n=18, 60%) for collecting and using sensitive PI. However, only about one-third of health code apps highlighted what constituted sensitive PI (n=11, 36.7%) or sufficiently described the necessity for processing such sensitive PI (n=11, 36.7%). Only 1 app explicitly required separate consent for processing sensitive PI.

In the PI storage and protection stage (mean 63.3%, SD 30.8%), the scoring rate of level-2 indicators varied slightly. The mean compliance rate of storage security was 65.8% (SD 32.6%). Most of the 30 apps outlined the level of technical security measures satisfactorily (n=27, 90%) and informed users about potential security risks (n=25, 83.3%). Moreover, around half of the apps (n=16, 53.3%) extended their security explanations to include organizational management measures, while only 11 (36.7%) policies discussed the PI security agreements or certifications obtained. As for security incidents (mean 60.0%, SD 35.9%), although a significant portion of apps were committed to notifying (n=23, 76.7%) and reporting security incidents (n=23, 76.7%), only 26.7% (n=8) of PI processors were committed to assuming legal responsibilities in the event of such incidents.

In the stage of PI sharing, transfer, disclosure, and transmission (mean 57.2%, SD 27.3%), the scoring rate of level-2 indicators varied substantially. As for public disclosure (mean 85.0%, SD 32.0%) in the privacy policies, we observed high compliance in specifying conditions for potential public PI disclosure (n=25, 83.3%) and requiring separate consent for such practices (n=26, 86.7%). These rates indicated a high degree of transparency and respect for user consent for public disclosure. The compliance rate of PI sharing and transfer (mean 53.3%, SD 32.4%) indicated a mixed landscape in terms of transparency and user consent in these practices. Most apps required separate consent for sharing or transferring PI (n=27, 90%), whereas only half of the apps described the methods of PI transfer (n=16, 53.3%) and types of PI involved in the transfer (n=15, 50%). Less than half of the policies disclosed the basic information about PI recipients (n=13, 43.3%) and safety precautions (n=11, 36.7%). Rules governing PI transfer during specific events were missing in 66.7% (n=20) of privacy policies. As for cross-border transmission (mean 53.3%, SD 38.6%), most apps specified storage locations of PI (n=22, 73.3%), while only one-third of apps mentioned compliance with relevant cross-border transmission laws (n=10, 33.3%). As for subcontracting PI processing (mean 46.7%, SD 28.7%), less than half of the apps required separate consent for these activities (n=14, 46.7%) or ensured supervision (n=14, 46.7%), mainly via signed agreements.

In the stage of PI deletion (mean 52.2%, SD 29.4%), most privacy policies of the 30 apps stated the PI retention period (n=23, 76.7%), with 2 mentioning that PI would be retained for more than 6 months. By contrast, the mean scoring rate of deletion and cessation was low (mean 40.0%, SD 27.1). Although most apps committed to PI deletion or anonymization after the retention period (n=22, 73.3%), only 2 (n=2, 6.7%) apps claimed to notify third parties to delete or cease processing PI.

Concerning individual rights (mean 59.3%, SD 25.7%), most of the 30 apps explained individuals’ various rights effectively, including the right to inquire (n=25, 83.3%), correct (n=24, 80%), and delete PI (n=24, 80%); cancel the account (n=21, 70%); withdraw consent (n=18, 60%); and request an explanation of the privacy policy (n=24, 80%). Providing this information empowered individuals to exercise their rights. However, only a few apps (n=4, 13.3%) recognized the right to obtain copies, and only 1 app explained the right to refuse business marketing using automated decision-making. In addition, a majority of the apps (n=21, 70%) listed exceptions for obtaining consent as provided by applicable laws or administrative regulations.

Concerning PI processor duties, we found a mean compliance rate of 43.7% (SD 23.8%). While many of the 30 apps provided methods for individuals to inquire (n=25, 83.3%), correct (n=24, 80%), and delete PI (n=24, 80%) as well as cancel their account (n=21, 70%) and withdraw consent (n=18, 60%), there was a significant shortfall in institutional oversight and risk management. Specifically, only a small percentage of PI processors appointed a PI protection officer (n=2, 6.7%) or conducted a PI protection assessment (n=2, 6.7%), with only 1 case assessing the purpose, method, impact, and protective measures for processing PI. Regular compliance audits were almost nonexistent (n=2, 6.7%). Furthermore, although many apps provided avenues for inquiries and complaints by disclosing contact information for requests (n=25, 83.3%) and means for complaints (n=15, 50%), only a minority of them committed to addressing these within 30 days or a legal time limit (n=19, 63.3% for requests and n=9, 30% for complaints). Only 3 apps explained the limitations of the use of automated decision-making in the information system.

## Discussion

### Principal Findings

In this study, we reviewed 30 privacy policies of health code apps in the mainland of China and assessed the compliance of these privacy policies with the PIPL and PI Specifications. Bardus et al [13] presented a systematic review of COVID-19 contact-tracing apps used worldwide to analyze apps’ approach to data protection and privacy. However, they only identified one health code app (ie, Alipay Health Code) on May 28, 2020, and found the privacy policy was not available, which excluded China from their scope. In addition, Ni et al [33] referred to the PI Specifications and developed a scale to evaluate the compliance of the privacy policies of China’s chronic disease apps. However, their study was conducted before the PIPL came into force, so their scale could not reveal the regulatory development and the mandatory requirement of the PIPL. Therefore, it is necessary to re-evaluate the compliance of the privacy policies of health code apps based on regulatory developments in China.

Our findings illustrated a mixed landscape of compliance status, revealing both areas of commendable adherence and notable gaps in the alignment with the legal framework. While 13 of the 30 apps scored below the mean average, a concerning 20% (n=6) scored under 40%, signaling the urgent need for improvements and highlighting potential threats to PI. When examining compliance across the information life cycle, we found that the highest alignment was in the realm of general attributes with a mean compliance of 85.6% (SD 23.3%). This indicates a prevalent transparency among PI processors regarding their basic information, service range, and privacy policy content and updates. However, 14 (46.7%) apps did not mark any updates after the PIPL came into force, raising concerns regarding the timeliness of policy updates in alignment with regulatory changes.

In the realm of PI collection and use, the compliance rate of sensitive PI protection (mean 51.4%, SD 26.0%) was significantly lower than the rate of general PI protection (mean 83.3%, SD 24.3%). Such discrepancy contradicts the special protection for sensitive PI as provided in the PIPL (in particular, section 2, chapter 2 of the PIPL) and may reduce users’ risk awareness. According to the PIPL, PI processors may process sensitive PI only when there is a specified purpose and sufficient necessity, and when stringent protective measures are adopted (article 28 of the PIPL). Meanwhile, PI processors should notify the users of “the necessity of processing such sensitive PI” and “the influence on the individual’s rights and interests” (article 30 of the PIPL), and obtain separate consent (article 29 of the PIPL). A majority of the 30 apps elaborated on the specific purpose (n=18, 60%) and influence of processing sensitive PI (n=24, 80%), and the various strict protective measures (n=19, 63.3%). In addition, 80% (n=24) of the apps ensured explicit consent for collecting the PI of minors. However, only 1 app required separate consent for processing sensitive PI, while the other 29 apps only obtained general consent for processing all types of PI. In addition, although 60% of the apps described the specific purpose for processing sensitive PI, fewer apps underscored the exact sensitive PI collected for the health code apps (n=11, 36.7%) and explained the necessity of processing such sensitive PI (n=11, 36.7%). As a result, users may not fully understand the specific sensitive PI involved, why such processing becomes necessary, and the exact content of their consent, in particular, whether the specific consent required for processing sensitive PI is implied or mixed with general consent.

While the overall mean compliance of PI storage and protection stood at a relatively satisfactory level (mean 63.3%, SD 30.8%), the depth and commitment underpinning this compliance varied significantly. Although a robust 90% (n=27) of the 30 apps explained their technical security measures and over 80% (n=25) informed users of potential risks, only 36.7% (n=11) of the assessed apps discussed or provided evidence of PI security agreements or certifications. This shortfall underscores the potential vulnerability of PI, considering that such certifications often serve as benchmarks for best practices. Equally concerning is the lack of commitment to legal responsibility, which is not compliant with articles 66 to 70 of the PIPL. While 76.7% (n=23) of the assessed apps pledged to notify and report security incidents, only 26.7% (n=8) of PI processors were explicitly committed to bearing legal responsibilities during such breaches. This discrepancy raises pressing questions about accountability and reinforces the need for stronger mechanisms that can assure users of adequate protection and remediation in the face of potential PI infractions.

In the stage of PI sharing, transfer, disclosure, and transmission (mean 57.2%, SD 27.3%), most of the 30 apps required separate consent for PI transfer (n=27, 90%) and public disclosure (n=26, 86.7%). However, less than half of the evaluated apps required separate consent for subcontracting practices (n=14, 46.7%) and were committed to supervising such practices (n=14, 46.7%). This is notably lower than the expected standards set out in article 23 of the PIPL, which may reduce users’ situational awareness concerning the flow and security of their PI. Even fewer apps disclosed the basic information of PI recipients (n=13, 43.3%), safety precautions (n=11, 36.7%), and rules of PI transfer during specific events (n=10, 33.3%). The resultant opacity diminishes users’ ability to grasp the full trajectory of their PI, undermining their trust and inhibiting informed consent. Another note of concern is that only 2 apps notified third parties to promptly delete PI or cease processing due to user request or other circumstances. Not doing so can lead to prolonged PI retention beyond necessity, heightening the risk of data breaches or misuse.

In the stage of PI deletion, roughly one-quarter of the 30 apps (n=7, 23.3%) did not mention their PI retention period, which is the minimum period necessary for achieving the purpose of processing according to article 19 of the PIPL. While this extended retention raises concerns about the purpose and implications of such a practice, another deficiency is evident in the deletion and cessation protocols. Even though a significant percentage (n=22, 73.3%) of apps asserted the deletion or anonymization of PI after the defined retention span, only 6.7% (n=2) made a proactive commitment to ensure that third parties were notified to delete or halt the processing of PI promptly. This lack of third-party engagement presents potential vulnerabilities in the comprehensive safeguarding of PI, especially when the use of third-party services is common.

When evaluating individual rights concerning their PI, we found that a predominant number of the 30 apps efficiently elucidated the diverse rights of users to inquire (n=25, 83.3%), correct (n=24, 80%), and delete their PI (n=24, 80%); request an explanation (n=24, 80%); lodge a complaint; withdraw their consent (n=18, 60%); and cancel their account (n=21, 70%). Additionally, most apps (n=19, 63.3%) elaborated on exceptions for obtaining consent as provided by laws and regulations. This comprehensive coverage underscores a commendable effort in empowering individuals to exercise their rights with exemptions of informed consent provided by law. However, only 13.3% (n=4) acknowledged the right to obtain PI copies, while this right is explicitly provided in article 45 of the PIPL. More concerning is the fact that only 1 app addressed the right to refuse business marketing through automated decision-making, while article 24 of the PIPL calls for transparency, fairness, and the right to receive an explanation and to opt out of such marketing. These results reflect and further highlight a broad acknowledgment of individual rights by most apps. These crucial rights might inadvertently hinder users from fully realizing their entitlements under PI protection norms.

As for the duties of PI processors, our findings revealed a stark incongruity between policy statements and tangible practices for ensuring PI protection. Notably, the overall mean rate of compliance regarding the duties of PI processors was only 43.7% (SD 23.8%), with the 30 assessed health code apps particularly falling short in critical areas of impact assessment procedures (conduct PI protection assessment: n=2, 6.7%; assess the purpose, method, and impact of protective measures: n=1, 3.3%), PI protection officer appointment (n=2, 6.7%), regular compliance audits (n=2, 6.7%), and complaint management (convenient means to lodge complaints: n=15, 50%; commit to responding within legal time limits: n=9, 30%; dispute resolution involving external parties: n=10, 33.3%). These key components are outlined in articles 50, 52, 54, and 55 of the PIPL. The deficiencies in impact assessment, for instance, can result in unforeseen risks or breaches as changes in technology or external threats evolve. The absence of dedicated PI protection officers indicates there is no designated authority to ensure that PI is handled in strict accordance with the law. Without regular compliance audits, apps may drift from best practices over time, unknowingly exposing PI to risks. Lastly, inadequate complaint management mechanisms not only breach the PIPL but also degrade user trust, leading to potential withdrawals from the app or caution against sharing sensitive PI.

### Recommendations

In 2020, during the phase termed “the people’s war against the epidemic” [39], the right to life and health, constituting public health, was prioritized over the protection of PI. This only changed when the PIPL came into force on November 11, 2021. Health code apps essentially compromised individual rights for necessary prevention and control of the COVID-19 pandemic, which in turn required PI processors to properly protect the use of PI. The once temporary and mandatory use of health code apps could be seen as a “trade-off” to win the war. However, the legitimacy of processing sensitive PI and the further retention of such PI have come into question, especially when PIPL was enacted. Once China eased its stringent zero-COVID policy [40], green health codes were no longer required for movement and travel [41].

In light of the evolving situation, China’s government faces a crossroads regarding the future of health code apps, where it must choose between shutting down its services or expanding their use. This choice calls for a balanced policy that considers both public health and PI protection.

First, it is essential to reconsider and clearly articulate the legitimacy of extending health code apps more broadly in public health services such that users are notified and their consent is separately obtained. Initially, the deployment of health code apps was an emergency response to prevent and control the pandemic by performing the statutory duties of government agencies. Because this rationale no longer holds, the continuation of intrusive surveillance through these apps should not be allowed without further evaluation [3]. It is inadequate to justify the routinization of health code apps based merely on general purposes such as providing convenience to citizens, protecting people’s health, or incorporating big data technologies in public health. Furthermore, the benefits and resources associated with health code apps do not sufficiently justify processing sensitive PI in their expanded use. Government agencies should encourage more discussions on the necessity and purpose of the continued use of health code apps to address the intensified concerns about data privacy, data security, and data governance as a whole [42].

Second, as China looks toward the normalization of health code apps, it becomes paramount to not only uplift the compliance level of relevant privacy policies but also fortify their enforcement mechanisms. Areas of improvement, based on the information life cycle, encompass enhanced clarity during PI collection and use, improved storage protection measures, more transparent sharing and transfer protocols, clearer deletion guidelines, and a broader acknowledgment of individuals’ rights accompanied by actionable exercising avenues. Moreover, PI processors should be diligent in their responsibilities and conduct regular audits and impact assessments. The COVID-19 pandemic not only necessitated and justified the intervention of health code apps during public health emergencies but also brought new challenges to PI protection in their broader application in the postepidemic era. Legal protections for PI seek to facilitate the processing of PI to achieve public benefits while still furnishing reasonable and sufficient protection [43]. Striking a nuanced balance between public interests and PI protection has become an important theme, requiring more effort and thought alongside the rapid development of big data technologies. Only through such concerted efforts can we ensure that these apps serve as not only functional tools but also vanguards of PI, fostering trust and confidence in their user base.

### Limitations

One limitation of this study is the lack of empirical evaluation of protective measures for processing sensitive PI. Technological equipment and expertise are necessary to assess whether the protective measures adopted by health code apps are stringent enough to prevent abuse and leakage of sensitive PI. We hope that technological professionals can engage in health code assessment and provide insightful research. Another limitation is that this study’s focus on the informed consent model does not address the gaps between the health code practices and best practices of contact-tracing apps. Further studies comparing both the existing and future practices of Chinese health code apps with other countries’ practices can use this study as a starting point.

### Conclusion

Health code apps are not only an innovation for monitoring and controlling public health emergencies such as the COVID-19 pandemic, but they can also act as a strategic health and medical service platform. Our analysis of 30 privacy policies sheds light on the multifaceted nature of compliance with the PIPL and related specifications. Although commendable strides have been made, significant gaps remain in pivotal areas of the information life cycle. These discrepancies not only pinpoint the exigence of robust PI protection measures but also underscore the importance of fostering trust among users. Only with sufficient PI protection can health code apps and other contact-tracing apps worldwide achieve the maximum value for both public and private interests.

## Supplementary material

10.2196/48714 Multimedia Appendix 1 Evaluation scale.

10.2196/48714 Multimedia Appendix 2 Health code apps and scoring rates.

## Abbreviations

PI: personal information
PIPL: Personal Information Protection Law
